# Treatment of eumycetoma with terbinafine alone and in combination with salvage therapy^⋆^

**DOI:** 10.1016/j.abd.2024.02.004

**Published:** 2024-08-07

**Authors:** Alexandro Bonifaz, Andrés Tirado-Sánchez, Denisse Vázquez-González, Javier Araiza, Luis Miguel Moreno-López, Gloria M. González, David Chandler

**Affiliations:** a Dermatology Service & Mycology Department, Hospital General de México “Dr. Eduardo Liceaga”, Ciudad de México, Mexico; b Internal Medicine Department, Hospital General de Zona 29, Instituto Mexicano del Seguro Social, Ciudad de México, Mexico; c Dermopathology Service, Hospital General de México “Dr. Eduardo Liceaga”, Ciudad de México, Mexico; d Department of Microbiology, Faculty of Medicine, Universidad Autónoma de Nuevo León, Monterrey, Mexico; e Dermatology Department, Brighton General Hospital, University Hospitals Sussex NHS Foundation Trust, Brighton, United Kingdom; f Department of Global Health and Infection, Brighton and Sussex Medical School, Brighton, United Kingdom

*Dear Editor,*

Mycetoma is a chronic disease that begins with the implantation or inoculation into the skin of microorganisms from soil and other sources. It is divided into two types according to etiology: eumycetoma, caused by filamentous fungi, and actinomycetoma, caused by aerobic filamentous bacteria.^1^, ^2^ It is considered a classic neglected and poverty-related disease, which is why the World Health Organization included it in the group of neglected diseases in 2016.^3^ Mexico is the country with the most reports of mycetoma after Sudan, although there are differences in the mycological profile.^4^, ^5^ The simplicity of mycetoma diagnosis contrasts with its treatment, especially for eumycetomas, since, antifungal agents are scarce and expensive in a disease that requires a minimum treatment period of one year. The first choice is oral itraconazole, which responds well in some cases, but cure rates remain low. The response depends on a number of factors, including the size and extent of the mycetoma, the possible involvement of bones, and the patient's health status.^2^, ^3^, ^6^ For these reasons, new effective and cost-effective therapeutic options should be sought. In particular, in those cases that do not respond to therapy with itraconazole, other treatments such as terbinafine alone or in combination should be tried.^7^

All cases here discussed were a confirmed diagnosis of eumycetoma, with observation of grains on direct examination, cultures (Sabouraud-dextrose agar), microscopic and molecular identification by PCR of the cultures obtained and skin biopsy. were performed for all of the cases. Cases that had failed therapy with itraconazole at therapeutic doses and for prolonged periods were included in the study, as were cases that experienced side effects or interactions with other drugs. Terbinafine doses varied from 250 to 750 daily, depending on disease severity. A complete blood count, liver function tests, renal function tests, and urinalysis were performed at the start of treatment and repeated every three months during treatment. Treatment success was evaluated clinically and by mycological examination, which included fresh examination and cultures to determine whether a complete cure or partial improvement had occurred.

Five patients were included in the study. The main demographic, clinical, mycological, and therapeutic data are shown in Table 1. Clinical and mycologic cure without relapse was achieved in 3 patients (60%) during follow-up up to one year after the last dose. Clinical improvement with significant tumor reduction was observed in two cases (40%) and no bone activity in one case (20%) (Fig. 1).

**Table 1:** Main demographic, clinical, mycological, and therapeutic data.

No	Age	Gender	Clinical location	Evolution (year)	Risk factor	Etiological agent	Bone damage	Previous treatment/Time	Reason for discontinuation	Treatment (doses). Terbinafine	Time (months)	Result
1	34	Male	Foot	4	None	*Fusarium chlamidosporum*	None	Itraconazole/3 months	Dyspepsia	500 mg/day	18	Cure
2	38	Male	Foot	12	None	*Madurella pseudomycetomatis*	Osteolysis	Itraconazole/4 months	Dyspepsia	500 mg/day	22	Improvement
3	32	Female	Foot	8	None	*Madurella pseudomycetomatis*	Osteolysis	Itraconazole/8 months	Poor response	750 mg/day 6 months	16	Improvement (No bone activity).
										500 mg/day 10 months		
4	44	Male	Hand	3	Diabetes type 2	*Biatriospora mackinnonii*	None	Itraconazole/1 month	Interaction with glibenclamide (Hypoglycemia)	750 mg/day 4 months	12	Cure
										500 mg/day 8 months		
5	41	Male	Foot	5	None	*Madurella pseudomycetomatis*	Osteolysis	Itraconazole/6 months	Poor response	Terbinafine 250 mg/day + Itraconazole 200 mg/day	20	Cure
								Terbinafine/4 months (independent)

**Figure 1:**
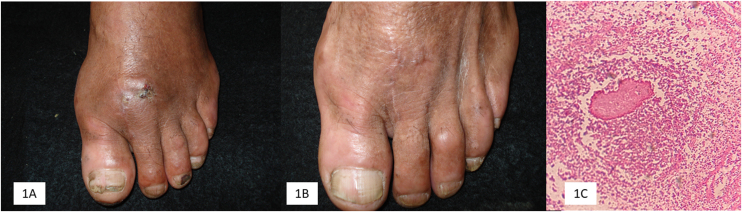
(A) Patient 1: Mycetoma due to *Fusarium chlamydosporium*, before treatment. (B) At the end of treatment 18 months. (C) Microabscess with granule in biopsy (Hematoxylin & eosin, 10×).

A series of 5 cases of eumycetoma treated with terbinafine was analyzed. A favorable response was observed, although a clinical and mycological cure was achieved in only three cases. In two patients (40%), itraconazole had been given at the correct dose and timing and had responded poorly, so a change in treatment was decided or due to side effects and drug interactions (dyspepsia and hypoglycemia), it is important to emphasize that terbinafine does not depend on the pH of its absorption, and its drug interactions are minimal, so it can be administered for a long time, necessary for chronic conditions such as mycetoma.^3^, ^7^

Terbinafine has moderate activity against mycetoma. In this series (Table 1), clinical and mycological cure was achieved in three cases (60%) with the use of terbinafine, in two cases as monotherapy (500 mg/day dose and the other with 750 mg/day and reduced to 500 mg/day), with a treatment duration of 16 and 18 months, respectively (Fig. 2). It is important to note that in our series, three patients had osteolytic activity and cure was achieved in only one of them; previously, treatment resistance has been observed in eumycetoma with bone involvement.^8^ N'diaye et al. from Senegal^8^ reported the response to treatment with terbinafine in 27 patients with eumycetoma with a dose of 1,000 mg/day divided into two doses over 24‒48 weeks. Another important experience with terbinafine was reported in Senegal by Sow et al.,^9^ who included 68 patients with eumycetoma of black grains who were also treated with terbinafine 1,000 mg, divided into two daily doses, in combination with surgical treatment. A clinical and mycological cure rate of 29.4% was achieved, better than itraconazole combined with surgery, which was 13%. Apart from the two earlier studies, other cases are sporadic in the literature.^10^

**Figure 2:**
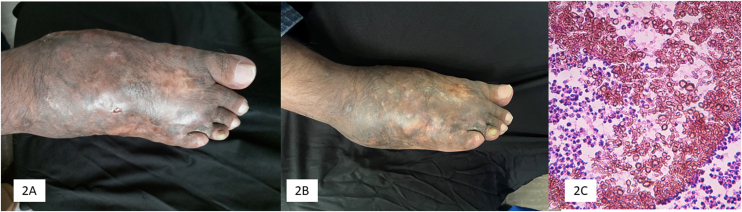
(A) Patient 5: Mycetoma caused by *Madurella pseudomycetomatis*, before starting treatment. (B) At the end of treatment. (C) Grain made up of thick, brown hyphae (Hematoxylin& eosin, 10×).

This work is a small case series evaluating the efficacy of terbinafine in the treatment of eumycetoma. The sample size here is too small to draw conclusions, but it shows an alternative treatment for evaluation in larger, comparative, and multicenter studies.

## Financial support

None declared.

## Authors’ contributions

Alexandro Bonifaz: Contributed to the writing and reviewing of the whole paper, was responsible for editing the manuscript, read and agreed to the published version of the manuscript.

Andrés Tirado-Sánchez: Contributed to the writing and reviewing of the whole paper, was responsible for editing the manuscript, read and agreed to the published version of the manuscript.

Denisse Vázquez-González: Contributed to the writing and reviewing of the whole paper, Clinical follow-up and control, review of the document, read and agreed to the published version of the manuscript.

Javier Araiza: Contributed to the writing and reviewing of the whole paper, histopathological studies, read and agreed to the published version of the manuscript.

Luis Miguel Moreno-López: Contributed to the writing and reviewing of the whole paper, Mycological and molecular studies and review of the document, read and agreed to the published version of the manuscript.

Gloria González: Histopathological studies, read and agreed to the published version of the manuscript.

David Chandler: Contributed to the writing and reviewing of the whole paper, was responsible for editing the manuscript, read and agreed to the published version of the manuscript.

## Conflicts of interest

None declared.
